# Increased Susceptibility of Radiation-Induced Intestinal Apoptosis in SMP30 KO Mice

**DOI:** 10.3390/ijms140611084

**Published:** 2013-05-24

**Authors:** Moon-Jung Goo, Jin-Kyu Park, Il-Hwa Hong, Ah-Young Kim, Eun-Mi Lee, Eun-Joo Lee, Meeyul Hwang, Kyu-Shik Jeong

**Affiliations:** 1 Department of Pathology, College of Veterinary Medicine, Kyungpook National University, Daegu 702-701, Korea; E-Mails: goomoonj@hanmail.net (M.-J.G.); 820jinkyu@hanmail.net (J.-K.P.); scarlet1999@hanmail.net (I.-H.H.); pretersensual@hanmail.net (A.-Y.K.); nikeun@hanmail.net (E.-M.L.); miffy525@hanmail.net (E.-J.L.); meeyul@hanmail.net (M.H.); 2 Stem Cell Therapeutic Research Institute, Kyungpook National University, Daegu 702-701, Korea

**Keywords:** SMP30, radiation, BAX, Bcl-2, intestine

## Abstract

Recently, senescence marker protein-30 (SMP30) knockout (KO) mice have been reported to be susceptible to apoptosis, however, the role of SMP30 has not been characterized in the small intestine. The aim of the present study is to investigate the role of SMP30 in the process of spontaneous and γ-radiation-induced apoptosis in mouse small intestine. Eight-week-old male wild-type (WT) mice and SMP30 KO mice were examined after exposure to 0, 1, 3, 5, and 9 Gy of γ-radiation. Apoptosis in the crypts of the small intestine increased in the 0 to 5 Gy radiated SMP30 KO and WT mice. Radiation-induced apoptosis and the BAX/Bcl-2 ratio in the SMP30 KO mice were significantly increased in comparison to each identically treated group of WT mice (*p* < 0.05). The levels of spontaneous apoptosis in both WT and KO mice were similar (*p* > 0.05), indicating that increased apoptosis of crypt cells of SMP30 KO by irradiation can be associated with SMP30 depletion. These results suggested that SMP30 might be involved in overriding the apoptotic homeostatic mechanism in response to DNA damage.

## 1. Introduction

Apoptosis is programmed cell death induced by a cellular “decision” to commit to a particular cell fate after cellular damage [1]. Controlled cell loss is essential for the removal of damaged cells, in particular, those whose survival may have the potential to promote the development of malignancies [2].

Radiation injuries result from the increased production of reaction oxygen species (ROS) such as superoxide anions, hydroxyl radicals, hydrogen peroxide, peroxyls, and alkoxy radicals. These reactive molecules may play an important role in the initiation and propagation of free radical chain reactions, inducing potentially severe damages to cells [3]. To maintain a protective redox balance, cells have evolved endogenous antioxidant defense mechanisms including non-enzymatic entities such as glutathione, ascorbic acid, uric acid, and also enzymatic ones such as catalase, superoxide dismutase, and glutathione peroxidase [4].

Senescence marker protein-30 (SMP30) is a 34 kD multifunctional protein highly expressed in mouse hepatocytes and renal tubular epithelial cells. Its expression decreases in an androgen-independent manner during aging and its absence in mice leads to a vitamin C deficiency [5–7]. Ishigami *et al.* reported that SMP30 has anti-apoptotic characteristics by demonstrating that hepatocytes of SMP30 KO mice showed an increased susceptibility to tumor necrosis factor-a (TNF-a) and Fas induced apoptosis in both *in vitro* and *in vivo* studies [8]. Matsuyama *et al.* also suggested that SMP30 plays a role as an anti-apoptotic factor by regulating Akt activity, and thus acts as a survival protein in hepatocytes [9]. Recently, several studies, including ours, have shown that SMP30 has many other important roles in the anti-aging and anti-apoptosis processes of various organs including the liver, bone, brain and lungs as well as skin tissue [10–15]. These results suggest that elucidating the role of SMP30 in normal and abnormal organs in pathological conditions may uncover a novel important factor in the mechanisms of aging-associated diseases.

Intestinal stem cells are prone to apoptosis caused by intentional or accidental radiation exposure because the intestinal epithelium is one of the fastest proliferating tissues in the body [16,17]. In the small intestine, apoptosis occurs rapidly (4 h) under physiological conditions and at the highest frequency in what are considered to be stem cells [18]. Recently, however, Hua *et al.* reported that intestinal crypt stem cells are relatively radioresistant attributed to the proficient use of DNA damage repair mechanism, which suggests there are more mechanisms that remain unknown and need to be elucidated [19]. Although the cell cycle of the intestinal epithelium is one of the fastest in the body, SMP30 has not been characterized in the small intestines which are especially subjected to apoptosis through metabolic perturbations or cytotoxic chemicals such as drugs, and irradiation, *etc.* γ-irradiation results in cell position-specific deletion through apoptosis of the crypt epithelial cells in the intestine [20]. Therefore, the aim of this study was to histopathologically investigate the role of SMP30 in spontaneous and γ-radiation-induced apoptosis in the mouse small intestines.

## 2. Results

### 2.1. Distribution of Apoptosis

**Figure 1:**
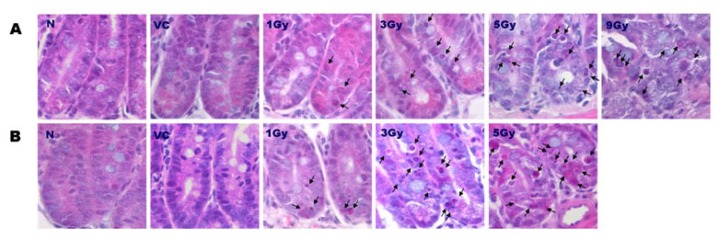

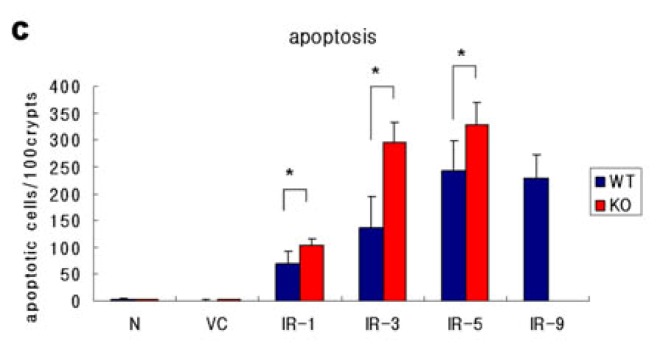
(**A**) Representative photomicrographs of intestines stained with hematoxylin and eosin (H & E) from WT mice. N, normal control; VC, vitamin C; Gy, Gray; Original magnification 1000×, Bars = 20 μm; (**B**) Representative photomicrographs of intestines stained with H & E from SMP30 KO mice. N, normal control; VC, vitamin C; Gy, Gray; Original magnification × 1000, Bars = 20 μm; (**C**) Apoptotic cells in the H & E stained sections were quantified. Values represent the mean ± SD in each group. WT, wild type; KO, knock out. * Statistically significant difference (*p* < 0.05).

**Figure 2:**
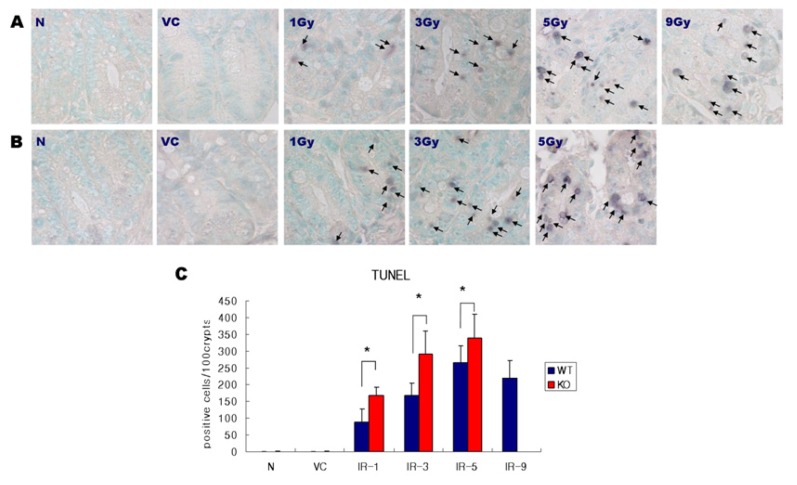
(**A**) Detection of apoptosis in the crypts of the intestine by terminal deoxynucleotide transferase labelling of DNA strand breaks (TUNEL) in the WT mice. N, normal control; VC, vitamin C; Gy, Gray; Original magnification 1000×, Bars = 20 μm; (**B**) Detection of apoptosis in the crypts of the intestine by terminal deoxynucleotide transferase labelling of DNA strand breaks (TUNEL) in SMP30 KO mice. N, normal control; VC, vitamin C; Gy, Gray; Original magnification × 1000, Bars = 20 μm; (**C**) Positive cells were quantified in the sections stained with the TUNEL method. Values represent the mean ± SD in each group. WT, wild type; KO, knock out. * Statistically significant difference (*p* < 0.05).

Following irradiation, typical morphological appearance of apoptosis in the crypts of the mouse small intestines was observed in both irradiated SMP30 WT and KO mice with hematoxylin and eosin (H&E) staining (Figure 1). As shown in Figure 1, there was no histological difference between 0 Gy N groups and VC groups of both WT and SMP30 KO mice showing only a normal structure and morphology of crypts. In WT mice, apoptotic cells (arrows) increased dose-dependently according to the increase in irradiation dose from 0 to 1, 3, and 5 Gy. In the 9 Gy group, no difference was observed compared to the 5 Gy group. Apoptosis of SMP30 KO mice (arrow) also increased dose-dependently according to the increase in irradiation dose from 0 to 1, 3, and 5 Gy. Radiation-induced crypt apoptosis was more significantly increased in the SMP30 KO mice compared with the WT mice at each dose of irradiation. Moreover, apoptosis in the crypt enterocytes was also observed by terminal deoxynucleotide transferase labelling of DNA strand breaks (TUNEL) assay (Figure 2), which positively detected cells containing DNA fragments (arrows). In the SMP30-WT mice, the number of apoptotic cells in the crypts rose with increasing radiation doses from 0 to 1, 3, and 5 Gy and with no further increases at 9 Gy of irradiation (Figure 1A,C). Radiation-induced apoptosis in SMP30-KO mice also increased in the same manner; however, it was much more dramatic compared to that of the SMP30-WT mice, especially at 3 Gy of irradiation. At 3 Gy of irradiation, the radiation-induced apoptosis of the small intestines in the SMP30-KO mice was more than 3 times than that of 1 Gy of irradiation in the SMP30-KO mice. When compared to 3 Gy of irradiation, there was no significant difference in the apoptotic index at 5 Gy of irradiation in the SMP30-KO mice (Figure 1B,C). As shown in Figure 2, the apoptotic index has a similar pattern shown by TUNEL staining.

### 2.2. Expression of BAX

**Figure 3:**
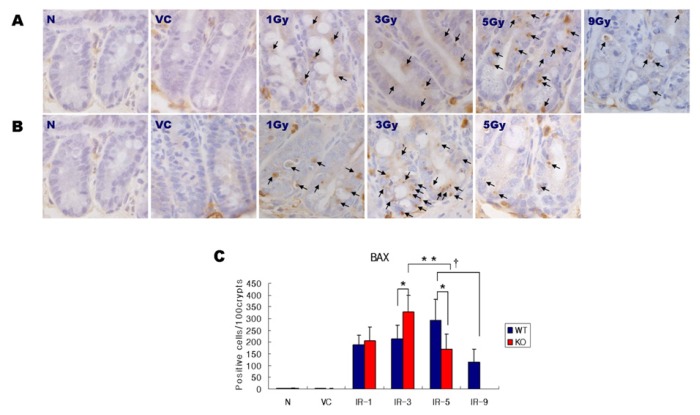
(**A**) BAX expression detected by immunohistochemistry in the crypts of the intestine in the WT mice: N, normal control; VC, vitamin C; Gy, Gray. Original magnification × 1000, Bars = 20 μm. (**B**) BAX expression detected by immunohistochemistry in the crypts of the intestine in SMP30 KO mice. N, normal control; VC, vitamin C; Gy, Gray. Original magnification 1000×, Bars = 20 μm. (**C**) Positive cells were quantified from immunohistochemically stained sections with BAX. Values represent the mean ± SD in each group. WT; wild type, KO; knock out. *, **, and †, Statistically significant difference (*p* < 0.05).

To assess whether SMP30 deficiency had an effect on the expression and localization of mitochondrial-associated apoptotic proteins, the number of BAX positive cells in the small intestines from each group of mice was quantified through immunohistochemical staining (Figure 3). In general, the intensity of BAX staining is positively correlated with the degree of DNA fragmentation (arrows). In WT mice, BAX protein was expressed at high levels in the crypt region and at lower levels in the lower part of the villi. The intensity of BAX was markedly stronger in the jejunum of the irradiated mice than that of the non-irradiated mice. The abundance of small intestinal BAX protein did not differ between the SMP30-KO and WT littermates in the non-irradiated groups.

The expression of BAX in the crypts was significantly increased after irradiation in both the SMP30-KO and WT mice. The increase of BAX expression in the SMP30 WT mice was dose-dependent when increasing the radiation dose from 0 to 1, 3, and 5 Gy. However, we found a significant decrease in BAX expression of the 9 Gy group compared that of 5 Gy group (*p* < 0.05). Morever, the BAX expression of the 9 Gy group also showed the lowest values among other radiation dose groups in the SMP30 WT mice. In SMP30 KO mice, the BAX expression showed an increasing trend in each 1 Gy and 3 Gy groups compared to those of the SMP30-WT mice; however, significant difference in BAX expression was observed only in the 3 Gy group. Interestingly, in the 5 Gy SMP30-KO group, there was a significant decrease in BAX expression when compared to the 5 Gy SMP30-WT group.

### 2.3. Expression of Bcl-2

**Figure 4:**
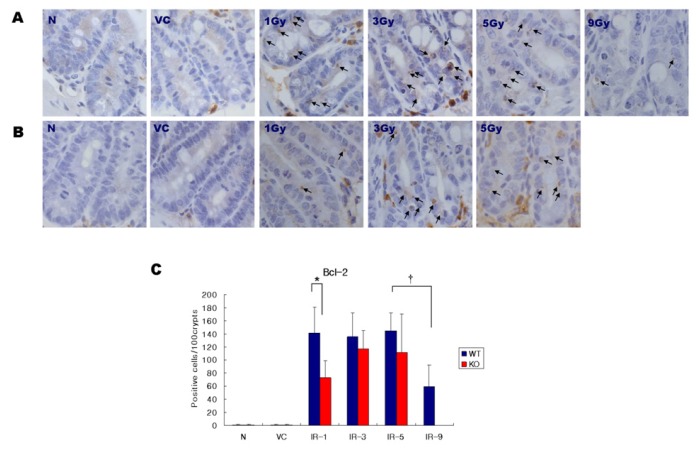
Bcl-2 expression detected by immunohistochemistry in the crypts of the intestine in WT mice (**A**) and SMP30 KO mice (**B**). N, normal control; VC, vitamin C; Gy, Gray. Original magnification × 1000, Bars = 20 μm. (**C**) Positive cells were quantified from the immunohistochemically stained sections with Bcl2. Values represent the mean ± SD in each group. WT, wild type; KO, knock out; * and †, Statistically significant difference (*p* < 0.05).

Figure 4 shows the expression pattern of the Bcl-2 protein in the base of the crypts taken from the small intestines of both the SMP30-KO and WT mice. There was no significant difference of the Bcl-2 expression level as determined immunohistochemically between WT and SMP30 KO mice of each N group and VC group, which only showed weak and diffuse reactions in some crypt cells. True positive expressions of Bcl-2 were more frequently observed within the crypts of small intestine in both irradiated WT and KO mice. In each of the 1, 3, and 5 Gy groups, the SMP30 KO mice showed more decreased Bcl-2 expression level compared with that of WT mice. Particularly, the gaps of Bcl-2 expression degree between WT mice and SMP30 KO mice were greatest at the 1 Gy groups. In WT mice, the increase of Bcl-2 expression induced by irradiation was constant up to 5 Gy, however, it was significantly decreased at the 9 Gy group compared with the other irradiation dose groups including the 1, 3, and 5 Gy groups. The difference in Bcl-2 expression between 5 Gy and 9 Gy was greater than twofold. These results suggest that irradiation can induce the increase of Bcl-2 expression levels in crypt cells.

### 2.4. The Ratio of BAX/Bcl-2

**Figure 5:**
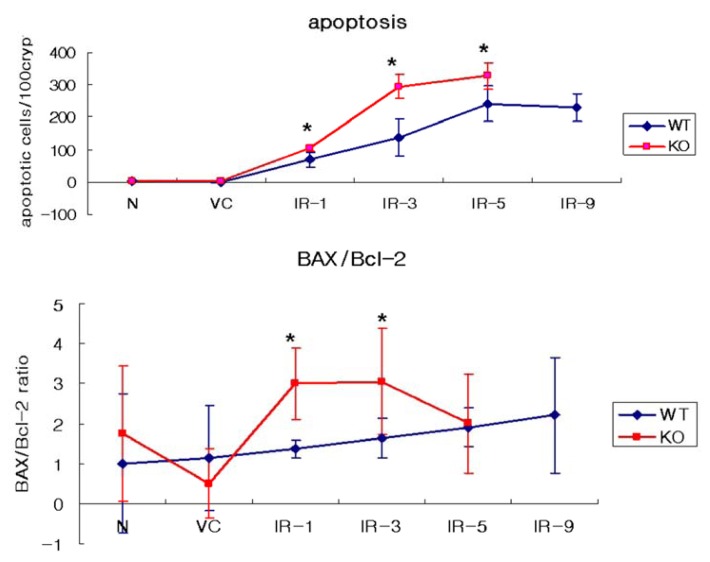
Comparison of apoptotic cells and the BAX/Bcl-2 ratio in the WT and SMP30 KO mice. The BAX/Bcl2 ratio significantly increased only in the 1 Gy and 3 Gy groups, while apoptosis increased in the 1 Gy, 3 Gy, and 5 Gy groups.

A comparison of apoptotic cells and the BAX/Bcl-2 ratio in the WT and SMP30 KO mice is shown in Figure 5. In the WT mice, a dose-dependent increasing trend observed in the data of the BAX/Bcl-2 ratio was comparable to the apoptotic index from the histomorphological assessment (Figure 5), although the increase in the apoptotic index was arrested in the group at 9 Gy group. The ratio of BAX/Bcl-2 was considerably increased in the 1 Gy and 3 Gy groups in the SMP30 KO mice compared to the WT mice. The induced apoptosis in the SMP30 KO mice was demonstrated by the ratio of BAX/Bcl-2 in the 1 Gy and 3 Gy groups.

## 3. Discussion

Radiotherapy is used in the multimodal treatment for neoplastic diseases in the pelvic abdomen. However, radiation unfortunately causes injury to highly dividing tissue including the easily affected small intestine of the pelvic region [21]. Following exposure to radiation, the appearance of apoptotic bodies increases at the base of the crypt as early morphological damage after radiation [22].

The aim of the present study was to determine the difference in spontaneous and irradiation-induced apoptosis between SMP30 KO and WT mice after abdomino-pelvic irradiation. Matsuyama *et al.* suggested that SMP30 plays a role as a survival factor by preventing apoptosis in the liver [9]. Since SMP30 KO mice cannot synthesize vitamin C due to the genetic disruption of gluconolactonase [10], it was also evaluated how the absence of vitamin C can affect spontaneous apoptosis in SMP30 KO mice. In this study, there was no significant differences in the degree of crypt cell apoptosis between vitatmin C treated SMP30 KO mice (VC group) and vitamin C depleted SMP30 KO mice (N group). The present study also showed no significant change in spontaneous apoptosis between non-irradiated SMP30 WT (WT N group) and KO (KO N group) mice. However, after irradiation, dramatically increased apoptosis in the SMP30 KO mice, compared to WT mice, was observed in each group irradiated with 1, 3, and 5 Gy. Moreover, all SMP30 KO mice irradiated with 9 Gy died suddenly before originally scheduled sacrifice time due to an excessive irradiation. Since this excessively high dose of irradiation can induce a cellular necrosis, which was unintended, the samples were excluded in the present study.

The expression of genes that regulate apoptosis, such as BAX, promoting apoptosis, and Bcl-2 promoting survival, play a key role in determining the survival threshold of cells and can determine cell survival or death after damage [23]. Oltvai *et al.* initially proposed the paradigm of the BAX and Bcl-2 interaction in the regulation of cellular apoptotic sensitivity [24]. In this present study, a remarkable increase in the a BAX/Bcl-2 ratio was observed in 1 Gy and 3 Gy groups in the SMP30 KO mice compared to that in the WT mice, as demonstrated by apoptotic index; however, the apoptotic index expressed as the number of apoptotic cells in the crypts did not exactly correlate to the BAX/Bcl-2 ratio in the present experiment. As the severity of cell injury caused by the irradiation increased, the apoptotic index seemed to hardly relate to the BAX/Bcl2 ratio. In the 9 Gy group in the WT mice, apoptosis reached the threshold, and late stage apoptotic cells, with severe DNA damage following phenotypic changes in the nuclei, hardly expressed BAX and Bcl-2. Thus, irradiation-induced apoptosis can be accelerated and completed earlier at higher Gy.

Interestingly, the shape of the graph for the SMP30 KO mice was shifted to the left compared to that of the SMP30 WT mice as shown in the apoptosis data in Figure 5. The static increase in apoptosis was observed in 5 Gy group in the SMP30 KO mice compared to that seen in the 9 Gy group in the WT mice, meaning that the SMP30 KO mice were more sensitive to stimulation by irradiation. The threshold was observed with 5 Gy of irradiation in the SMP30 KO mice and 9 Gy in the WT mice. By contrast, the results imply that the SMP30 protein has a possible role in cellular survival during irradiation-induced apoptosis. There are several possibilities in the role of SMP30 in irradiated-crypt cells. To clarify the specific role of SMP30 in apoptotic crypt cells, several issuess remain to be investigated. First, there is a possibility that SMP30 can protect crypt cells directly from irradiation-induced apoptosis. To elucidate this, it is essential to confirm if SMP30 is present in crypt cells, though we could not confirm this in the present study. Second, the protective effect of SMP30 in irradiated crypt cells can be induced by anti-oxidant effect of vitamin C biosynthesized by the SMP30 protein itself in the liver [13]. Moreover, to date, there have been several controversies regarding whether the death of crypt cells are induced directly by radiation-induced damage or indirectly by radiation-induced death of the endothelial cells forming the vessels of the villi [25]. Therefore, to verify a more specific role of SMP30 in irradiated crypt cells, additional experiments are necessary. We are also planning to continue experiments using isolated irradiated crypt cells of WT and SMP30 KO mice. Here, we report for the first time that SMP30 may be a key protein in the protection of the small intestines from injury inducing apoptosis. The absence of the SMP30 results in the promotion of apoptosis when intestinal epithelial cells are exposed to irradiation.

## 4. Experimental Section

### 4.1. Animal Models and Experimental Design

SMP30 KO (SMP30-knock-out) mice with a wild type (WT) C57BL/6 background and SMP30 (C57BL/6) were used. Eight-week old male SMP30 WT mice (*n* = 40), purchased from the Central Lab. Animal Inc. (Seoul, Korea), and SMP30 KO mice (*n* = 28), kindly provided by the Tokyo Metropolitan Institute of Gerontology (Tokyo, Japan), were kept under standard laboratory conditions with a 12-h light and 12-h dark cycle. Animals were given a vitamin C free diet (PicoLab^®^ Rodent Diet 20, Purina Mills Inc., Gray Summit, MO, USA) with vitamin C (Sigma, St Louis, MO, USA) in the tap water *ad libitum*. All experiments were performed in accordance with strict compliance to the National Institute of Health’s (NIH) guidelines for the care and use of laboratory animals. The mice were randomly divided into six experimental groups as shown in Table 1.

### 4.2. Irradiation

The whole bodies of 68 animals were exposed to single dose of 60Co gamma irradiation at 0, 1, 3, 5, and 9 Gy (Gray. 1 Gray is the absorption of 1 joule of radiation energy by 1 kg of matter. 1 Gy = 1 J/kg = 1 m^2^·s^−2^) at an exposure rate of 3.2 Gy/minute using a Gamma-cell Elan 3000 (Nordion International, Ottawa, ON, Canada) at the Korea institute of radiological and medical sciences. Each mouse was restrained in a specially designed close-fitting Perspex box (22 cm × 11 cm × 4 cm) and irradiated by fast neutron energy generated by a cyclotron. Mice in each group were sacrificed by deep anesthesia at 12 h after irradiation, in order to observe the histopathological changes because biological damage was time-dependent and maximal at 12 h after irradiation in our previous study [26].

### 4.3. Histology and Assessing Apoptosis

The small intestine was resected and immersed in a 10% neutral-buffered formalin solution and embedded in paraffin for histopathological analysis under light microscopy, which was done by three blinded pathologists. Four micrometers sections were then stained with hematoxylin and eosin (H & E). Identification of apoptosis was confirmed with a Terminal deoxynucleotidyl transferase-mediated nick end-labeling (TUNEL) technique that stains the oligofragmented DNA characteristically found in apoptotic nuclei [27] using a commercial apoptosis detection kit (Roche Diagnostics GmbH, Penzberg, Germany). One-hundred crypts per slide from the complete intestinal crypts that had been cut into transverse planes were scored for apoptosis. The incidence of cell death in the small intestine was quantified by counting the number of dead cells in each crypt with the H & E-stained sections at × 400 magnification by light microscopic analysis. In the small intestines, the base of the crypt was identified by the presence of Paneth cells. The distinctive morphological features of apoptosis, described in detail previously, were used to recognize apoptotic cells [28,29]. Small clusters of dead cell fragments were assessed as originating from one cell and given a single count and any doubtful cells were disregarded [30,31].

### 4.4. Immunohistochemistry

Small intestines, sectioned, were assessed by a routine immunohistochemistry method, using the monoclonal mouse antibodies of Bcl-2 (SantaCruz Biotechnology, Dallas, TX, USA) and BAX (Zymed, San Francisco, CA, USA). After deparaffinization and washing in 0.01M phosphate buffered solution (PBS), endogenous peroxidase activity was blocked by incubating the sliced sections in a 3% hydrogen peroxide in methanol for 30 min at room temperature and then the slides were microwave-treated for antigen retrieval. Sections incubated with primary antibodies were incubated with the appropriate biotinylated secondary antibodies on the following day. Immunoreactive materials were visualized with avidin-biotin-peroxidase complex solution using an avidin-biotin complex (ABC) kit (Vector Laboratories, Burlingame, CA, USA) with 3,3-diaminobenzidine (Zymed Laboratories Inc., San Francisco, CA, USA) according to the manufacturer’s instructions. Immunopositive cells were counted with at least 100 crypts per animal at 400× magnification by light microscopic analysis, and the average value was calculated.

### 4.5. Statistical Analysis

Results are expressed as the means ± SD. Paired Student’s *t* test was used to determine the significance of the differences between mean values of the experimental groups. The level of significance was set as a *p* of <0.05. Analysis of variance (ANOVA) was used to compare the differences between the multiple groups.

## 5. Conclusions

In conclusion, SMP30 protects intestinal cells from apoptosis, and the absence of this pro-survival protein is associated with alterations in anti- and pro-apoptotic molecules. To date, numerous studies on the SMP30 protein have made an effort to demonstrate the function of the protein in the body. Although the precise mechanism of the anti-apoptotic effect of SMP30 in the small intestine has yet to be revealed, we expect that the present study will aid in elucidating the role of SMP30 in apoptosis and help in developing therapeutic strategies after irradiation in humans.

## Figures and Tables

**Table 1:** Group and treatment protocol of SMP30-WT and SMP30-KO mice.

Group	Treatment	SMP30-WT	SMP30-KO
N	Vit.C-free diet + tap water	6	5
VC	Vit.C-free diet + Vit.C 2.5mg/ml in drinking water	6	5
IR-1 Gy	Vit.C-free diet + tap water + IR-1 Gy	7	6
IR-3 Gy	Vit.C-free diet + tap water + IR-3 Gy	7	6
IR-5 Gy	Vit.C-free diet + tap water + IR-5 Gy	7	6
IR-9 Gy	Vit.C-free diet + tap water + IR-9 Gy	7	

Vit.C, Vitamin C; IR, Irradiation type ^60^Co-gamma irradiation. Vitamin C-free diet was administered for 4 weeks before irradiation.
